# Impact and Influence of a Teaching Resident Rotation on Emergency Medicine Resident Physicians

**DOI:** 10.1002/aet2.70034

**Published:** 2025-03-31

**Authors:** Catherine Yu, Rebecca Bavolek, Luigi Varilla, Jaime Jordan, Steven Lai

**Affiliations:** ^1^ Department of Emergency Medicine Rutgers Robert Wood Johnson Medical School New Brunswick New Jersey USA; ^2^ Department of Emergency Medicine David Geffen School of Medicine at UCLA Los Angeles California USA; ^3^ David Geffen School of Medicine at UCLA Los Angeles California USA

## Abstract

**Background:**

The Accreditation Council for Graduate Medical Education requires residency programs to train their residents to be teachers. Teaching resident (TR) rotations in emergency medicine (EM) residency programs provide both an opportunity to train residents in teaching skills and a dedicated teaching service for junior learners in the clinical setting. The impact that this experience has on the residents themselves is unknown. We sought to explore the impact of our residency program's TR rotation on our recent graduates.

**Methods:**

We conducted a qualitative study using semistructured interviews. We recruited our residency program's recent graduates and interviewed participants over a videoconferencing platform. We used a constructivist paradigm to guide our thematic analysis.

**Results:**

We interviewed 11 graduates and identified major themes regarding how the TR rotation impacted their comfort and preparedness to teach and supervise learners postgraduation: discovery of their teaching identity, communication skills, development of teaching and supervisory skills, and professional development.

**Conclusions:**

EM residents found TR rotations helpful in developing skills that prepared them to educate learners and supervise patient care postgraduation. The findings of this study may inform the use and development of TR rotations in EM and other specialties.

## INTRODUCTION

The Accreditation Council for Graduate Medical Education (ACGME) requires emergency medicine (EM) residency programs to train their residents to be impactful teachers.^1^ Demonstrations of the abilities to teach different audiences and to evaluate teaching effectiveness are core competencies in EM.^1^ Additionally, residents are required to engage in scholarly activity during their training and teaching activities are recognized by the ACGME as a form of scholarship.^1^ To address these requirements, some residency programs utilize resident‐as‐teacher (RAT) curricula to develop the teaching skills of their residents.^2^ While these interventions have been shown to be effective in improving residents’ teaching skills, there is wide variation in the content, length, and evaluation techniques among the open‐access curricula available.^3^ Furthermore, many RAT curricula are classroom‐based, which can limit the applicability of knowledge and skills learned to the clinical setting.^2^, ^4^

The emergency department (ED) environment itself poses challenges when balancing patient care and quality clinical education. The pressure of managing multiple patients while optimizing throughput as well as the prioritization of resuscitating acutely ill patients over other tasks can significantly limit teaching opportunities and negatively impact learning.^5^ There is concern that ED overcrowding further compounds this effect, though studies have revealed mixed results on the impact of ED crowding on education.^6^ EM residents are affected by these challenges not only as learners in the ED but also as teachers to junior residents, medical students, and other staff.

To combat these challenges and improve education for all learners in the ED, some residency programs have implemented teaching resident (TR) rotations that function both as a dedicated teaching service for learners and as an opportunity for residents to develop teaching skills. During these rotations, residents educate and supervise the patient care of junior learners in the clinical setting but typically do not have primary patient care responsibilities. There are limited published data on the outcomes of this type of educational intervention; however, one program found that adding dedicated TRs to their ED improved learning, procedure performance, and patient care as perceived by medical students, resident learners, and attending physicians.^7^ To our knowledge, there have been no studies to date on the impact of the role on the TRs themselves, specifically how the experience prepares them to teach and supervise learners as attending physicians.

The purpose of this study was to explore the perspectives of recent graduates on the TR rotation experience as attending physicians postgraduation. This information can provide important insights to inform the use and development of TR rotations both inside and out of EM.

## METHODS

### Study design

We conducted a qualitative study using semistructured interviews with our residency program's recent graduates through a common videoconferencing platform. We analyzed the data inductively using thematic analysis with a constructivist paradigm.^8^, ^9^ This study was certified as exempt research by our institution's institutional review board.

### Study population and setting

We recruited our residency program's two most recent classes of graduates at the time of study, the classes of 2022 and 2023, through email invitations. We interviewed the 2022 graduates during the summer of 2023 and 2023 graduates in the fall of 2023. We used a common online videoconferencing platform to conduct the interviews. One member of the study team (CY) conducted the individual interviews, which lasted around 15 minutes each. Each interview was recorded with the participant's permission, transcribed verbatim by members of the study team (CY and LV), and deidentified prior to analysis.

### Instrument development

Our author team of education researchers and residency program leadership developed the interview script after we reviewed the literature and did not find an instrument applicable to this study. The script included the following elements: (1) introductory questions regarding current professional role, work setting, and previous teaching training and experience; (2) description of learners, teaching responsibilities, and teaching challenges in current role; (3) impact of the TR rotation on preparedness, motivation, and confidence to teach in current role; (4) specific elements of the TR experience applied in current role; (5) influence of the TR rotation on job or fellowship search and career goals during residency; and (6) suggestions for improvement for the TR rotation at our residency program. Most of the questions developed were open‐ended to maximize depth of response. Prior to administration on study participants, we piloted the instrument on a small sample of representative subjects composed of fellows and junior faculty to optimize response process validity. We made minor changes to question length and terminology to improve the clarity and understanding of questions based on their feedback. The interview script is available as supplemental material accompanying the online article.

### Teaching resident rotation

Our residency is a four year academic/university hospital program in which all fourth‐year residents participate in the TR rotation. Teaching shifts are scattered longitudinally throughout the year during ED blocks. The TR supervises and teaches first‐ and second‐year residents as well as medical students. All the fourth‐year residents are given an orientation to the TR role in the beginning of the academic year in which they are also taught an introduction to different teaching strategies.

### Data analysis

Two members of the study team (CY and LV) independently reviewed and coded the deidentified interview transcriptions line by line using the constant comparative method until thematic saturation was achieved.^10^ The two researchers subsequently met to reach consensus on the generated codes and to develop any new codes that were missed initially. The researchers further refined the codes into themes using the constant comparative method.^10^ When discrepancies arose, additional members of the study team (SL and RB) reviewed the findings and achieved negotiated consensus through in‐depth discussion.

### Reflexivity

Our author team consists of researchers from the same academic institution during the time of study and most members of the group were involved in residency program leadership. We were mindful that these shared traits, along with our close professional relationships with our recent graduates, could lead to similar interpretations of the data. To address this, members of the author team met regularly to discuss the ongoing research process and reflect on the available results. Additionally, the primary author (CY) met regularly with a research mentor and a group of peers outside of the author team to reflect on the theoretical frameworks, goals, and interests of this study.

## RESULTS

We invited all 30 graduates from the classes of 2022 and 2023 for the study and 13 agreed to be interviewed. Thematic saturation was achieved after the 11th subject. Six participants were in the class of 2022 and five participants were in the class of 2023. Three participants identified as female and eight identified as male. The ages of the participants ranged from 30 to 40 years.

Of the 11 participants, seven were at academic/university hospital sites, two were at community/academic‐affiliated sites, and two were at community sites. Five of the seven participants working at academic/university hospital sites were completing fellowships. The two participants working at community/academic‐affiliated sites worked with EM and non‐EM residents. The two participants at community sites had no learners.

### Graduates’ perspectives on the impact of the TR rotation on preparedness to teach/supervise

The perspectives of the participants regarding the impact of the TR rotation on their comfort and preparedness with teaching and learner supervision post‐graduation are highlighted in Table 1. We identified five major themes: discovery of teaching identity, communication skills, development of supervisory skills, development of teaching skills, and professional development.

**TABLE 1:** Graduates’ statements on the impact of the TR rotation on preparedness to teach/supervise.

Theme	Representative excerpts
Discovery of teaching identity	“I got to play around with different teaching and supervisory styles during the rotation which helped prepare me in my current job.” “The extra time I had with my learners during my [TR] shifts allowed me to teach beyond just the medical science. It included systems, efficiency, patient rapport, and even medical‐legal aspects of medicine.” “Teaching is a much different set of skills than just doing [the work by yourself]. It takes a lot of patience. I think during the teaching role, I realized I like doing things myself rather than watching others do and sometimes fail at doing.”
Communication skills	“There are specific [teaching methods] that learners found helpful that I did as a TR that I've tried to continue as an attending, which I feel helps bolster the relationship I have with the learners and opens up pathways for communication.” “Learning to give feedback was a huge part of the experience as many learners would ask for it directly. I also like to continually ask for feedback, like ‘was that helpful for you?’”
Development of supervisory skills	“[The rotation] taught me which types of patients or complaints I need to oversee more carefully … and what to do in terms of whether I should eyeball a patient first vs. which ones [the learners] can go see first.” “I've learned ways to offer my help when I see that the department is getting busy and the residents are getting overwhelmed, for example, asking residents ahead of time if they'd like it if I helped put in orders for them or another xyz task. Also knowing when to step back from helping with these clinical tasks and ensuring that they are learning how to do those tasks on their own and efficiently.”
Development of teaching skills	“The TR shift helped solidify having to shift gears in the level of complexity of the discussions depending on who my learner was. So I would have a different discussion about an illness with a medical student than with an intern, and I've used that model to reframe some of my discussions with patients now which has been really helpful.” “I think the rotation taught me how to dig deeper with learners and figure out what they don't know. For example, asking follow‐up questions to their assessment and plan because I want them to articulate their reasoning behind their decision and think the case through.” “The rotation strengthened my ability to convey information, such as learning to teach and supervise procedures, which is a whole different skill—making sure you are prepared beforehand and giving more subtle or nonverbal cues to the learner if they should do something differently since you are in front of the patient.”
Professional development	“I think the [TR] rotation definitely got me used to working with learners and prepared me to think about that additional layer of ‘where is the educational potential here?’ and ‘how do I give someone enough autonomy so that they can learn?’” “The experience made you review and examine your own medical knowledge. I would come in prepared with a couple notecards to talk about a topic if there were an opportunity. I was only able to give these talks a number of times based on patient volume, but it was nice to have the discipline of preparing small discussion points you could have ready to go.”

Abbreviation: TR, teaching resident.

#### Discovery of teaching identity

Participants identified several ways in which the TR rotation helped them explore aspects of their teaching identity, such as experimenting with different teaching styles and appreciating the breadth of medical education beyond just the basic sciences. Many participants reported reflecting on their own learning styles during their TR rotations to try and better understand the perspectives of their learners.

#### Communication skills

Participants reported that the TR rotation was beneficial in practicing their communication skills which ultimately helped build productive relationships with their learners. All participants noted how the TR rotation was helpful in learning how to give and receive feedback.

#### Development of supervisory skills

Participants appreciated the opportunity to practice and hone their supervisory styles while in the TR role, specifically learning how to balance clinical and teaching duties. Participants also reflected on their experiences navigating the complexities of efficient patient care while allowing for appropriate learner autonomy without being overbearing.

#### Development of teaching skills

Participants identified several ways in which being the TR helped develop their teaching skills, such as learning to tailor to different levels of learners and recognizing their limitations. Some participants reflected on their appreciation for the advanced skill set needed to teach and supervise procedures.

#### Professional development

Lastly, participants noted that having the TR experience in their last year of residency was beneficial in their transition to becoming new attending or fellow physicians. Many participants also commented on how being in the TR role improved their own medical knowledge.

### Graduates’ perspectives on the influence of the TR rotation on career direction

All graduates noted that the TR rotation did not ultimately influence or change their career directions or choices during residency. The perspectives, along with representative quotes, are summarized in Table 2. The seven graduates who went on to academic practice reported that the TR rotation reinforced their chosen career path. Many participants had prior teaching experiences and they noted that the TR rotation helped confirm their continued interest in teaching and working with learners postresidency.

**TABLE 2:** Graduates’ statements on the influence of the TR rotation on career direction.

Representative excerpts
“By the time I began the TR rotation I had already decided I was going to do academics from prior experiences of enjoying teaching. The experience confirmed it for me in the clinical setting.” “While I'm not at an academic site now, I may potentially want to be at one later, and since I really enjoyed the teaching rotation, it made me think that I would have fun teaching residents … all in all I would not say that [the TR rotation] completely changed any career decisions while I was in residency, but it did open my mind to opportunities in the future.” “I wouldn't totally discount [a teaching/academic role in the future], but it would be at least a decade or so. At my site, I love my midlevel providers, I love the ancillary staff, the patient population. And I do not think the pace is too difficult… right now, I am happy with where I am at.” “I feel like the teaching resident shift was a pertinent negative for me. I was really considering academics and I thought that maybe it would be a good fit for me. I found that doing the teaching residents shifts—I enjoyed them less than my regular shifts. What I was missing was the engagement of myself with the patient and really getting to know and feeling like I knew the nitty‐gritty and the details of the story, building the rapport, doing the procedures myself, and having more control over how the case went. I found those were really important to me. I liked teaching the learners, but I felt like those shifts were longer than my other shifts.”

Abbreviation: TR, teaching resident.

Among the four graduates who went on to community and community/academic‐affiliated practices, three reported that while the experience did not change their career direction during residency, their exposure to academic medicine through the TR rotation opened their minds to potentially transitioning to an academic or academic‐affiliated practice in the future. One of the four graduates noted that the rotation turned them away from pursuing an academic career down the line, citing their lack of engagement and control over aspects of patient care as downsides to being in the TR role.

## DISCUSSION

This study provides key themes on the impact and influence of TR rotations on residents’ teaching skills and professional development. The insights from our study reflect how clinical teaching opportunities like the TR role can fulfill the ACGME requirement for EM residency programs to train their residents to be impactful and effective teachers.

The TR rotation is unique in that it allows senior residents to develop teaching and supervisory skills while functioning as a dedicated teaching service for junior learners in the clinical setting. While research has shown that learners benefit from TR programs, our study adds to the literature on how the resident teachers themselves benefit from the role. The participants in our study highlighted their appreciation for the opportunity to discover their own style of teaching and supervising, such as learning to tailor to different levels of learners as well as finding the appropriate balance between clinical and teaching duties. Research on other educational interventions in EM such as RAT programs has shown similar impacts on self‐assessed teaching ability and confidence among participating residents.^11^, ^12^ Participants in our study also discussed how the rotation impacted their professional development in that it allowed them to get used to communicating and working with learners, thus preparing them for their roles as attending or fellow physicians postresidency.

The TR rotation can also give senior residents the opportunity to experience what a career in EM could look like working with learners after residency. Exposure to different career paths through formalized curricula, along with mentor support and personal interest in an area, have been noted to be major factors in resident career choices.^13^ While none of our participants reported a change in ultimate career decision based on their TR experience alone, they noted that the exposure to academic EM through the TR role was valuable for their professional development. Those who were already planning on academic practice reported that the experience was a positive reinforcement on their career choice and those who went on to practice in either community or community/academic‐affiliated settings had mixed reception toward academic medicine after the experience. The timing of the TR rotation at our program occurs during the resident's last year of training, which is likely too late to evoke a major change in career choice. Perhaps earlier implementation of the rotation or exposure to other teaching and academic experiences throughout residency could result in different outcomes.

Future studies should investigate an objective measure of skill development from TR rotations to better understand their value. Future directions should also examine the impact of similar TR rotations at other residency programs. The perspectives of other stakeholders, such as residency program leadership, could provide additional insight and important discussion on how to improve the rotation for the residents. Furthermore, multi‐institutional collaboration in the development of a set of best practices for TR rotations could benefit programs with existing rotations as well as programs looking to implement one at their site.

## LIMITATIONS

This study has limitations that must be considered in interpreting our findings. We interviewed a small sample of graduates from a single residency program. We may have missed important perspectives from graduates who were not interviewed and the results may not be generalizable to residency programs in other training environments. Our interview format was mostly open‐ended and participants were encouraged to speak freely; however, it is possible that not all relevant questions were asked. Though we purposefully recruited the program's two most recent classes of graduates at the time of study to minimize recall bias, there likely remains an element of this bias which impacts the findings. Since one class was interviewed one year after graduation while the other class was interviewed only a few months after, this time difference may have impacted the results. Interview studies are also limited by several response biases such as courtesy bias and social desirability bias. Despite these limitations, we believe this study provides important insights into the impact of a TR rotation.

## CONCLUSIONS

Emergency medicine residents in our study found teaching resident rotations valuable in the discovery of their teaching identity, communication skills, development of teaching and supervisory skills, and professional development. These findings may inform the use and development of teaching resident rotations in emergency medicine and other specialties.

## AUTHOR CONTRIBUTIONS

Catherine Yu: Conceptualization; investigation; writing—original draft; methodology; validation; visualization; writing—review and editing; formal analysis; project administration; data curation; supervision; resources; software. Rebecca Bavolek: Conceptualization; methodology; validation; visualization; supervision; project administration; writing—review and editing; resources. Luigi Varilla: Writing—review and editing; data curation; formal analysis; software. Jaime Jordan: Writing—review and editing; visualization; resources. Steven Lai: Supervision; methodology; validation; visualization; writing—review and editing; conceptualization; resources.

## CONFLICT OF INTEREST STATEMENT

The authors declare no conflicts of interest.

## Supporting information


Data S1.

## Data Availability

The data that support the findings of this study are available on request from the corresponding author. The data are not publicly available due to privacy or ethical restrictions.
