# Supplementary material for: Impact and Influence of a Teaching Resident Rotation on Emergency Medicine Resident Physicians
Source: AEM Educ Train. 2025 Mar 31;9(2):e70034. doi: 10.1002/aet2.70034 (PMC11957947; doi:10.1002/aet2.70034)
Supplement: Supplementary file 1 — Data S1. [file AET2-9-e70034-s001.pdf]

Core Interview Questions

Impact of TR rotation post-graduation:

1. Please describe your current work setting (community, academic, county, mix)
2. Tell me about your role as a teacher in your current work environment.
    a. What are your teaching duties and responsibilities in your current work?
    b. Who are your learners? (EM residents, not-EM residents, medical students, PA students, nurses, etc)
3. What are the teaching challenges you face in your current work setting?
4. What was the TR experience in residency like for you?
5. How did the TR rotation impact your motivation to teach in your current work?
6. How did the TR rotation impact your confidence in skills as an educator?
7. In what ways did the TR rotation prepare you (or not) to teach in your current professional role?
8. Can you describe some examples of how you've applied specific elements of the TR curriculum and/or experiences in your work?
9. Which part(s) of the TR rotation were most impactful to developing your skills as an educator?
10. How did the TR help you overcome challenges as a teacher in your current work setting?

Influence of TR rotation on career:

1. How did your experience during the TR rotation influence your job or fellowship search during residency?
2. In what ways did the TR rotation impact your perceived ability to obtain a desirable job following residency?
3. How did your experience during the TR rotation influence your ultimate career choice?
4. In what ways (if any) did the TR rotation influence your future career trajectory?
5. In what ways (if any) did the TR impact your career goals?
6. In what ways (if any) did the TR influence your professional identity?
